# Engineering membrane‐bound alkane monooxygenase from *Marinobacter* sp. for increased activity in the selective ω‐hydroxylation of linear and branched aliphatic esters

**DOI:** 10.1002/pro.70511

**Published:** 2026-03-17

**Authors:** Jelena Spasic, Andrea Nigl, Huijin Cheon, Christine L. Kaiserer, Stela Galušić, Elske van der Pol, Lenny Malihan‐Yap, Jin‐Byung Park, Robert Kourist

**Affiliations:** ^1^ Institute of Molecular Biotechnology Graz University of Technology Graz Austria; ^2^ Department of Food Science and Biotechnology Ewha Womans University Seoul Republic of Korea; ^3^ Institute of Organic Chemistry Graz University of Technology Graz Austria

**Keywords:** alkane monooxygenase, enzyme engineering, oxyfunctionalization, polymer precursors, regio‐ and stereoselective hydroxylation

## Abstract

The regio‐ and stereoselective hydroxylation of unactivated C(sp^3^)‐H bonds is an important reaction in organic synthesis. While bacterial alkane monooxygenase AlkB catalyzes the terminal hydroxylation of aliphatic esters with excellent regioselectivity, the molecular principles of substrate recognition and selectivity of this integral membrane enzyme are still poorly understood. In this study, we investigated the substrate scope and engineered the medium‐chain alkane monooxygenase from *Marinobacter* sp. (M_AlkB) for the terminal hydroxylation of linear and branched esters of fatty acids and alcohols. For the first time, we demonstrated the stereoselectivity of AlkB toward prochiral substrates containing terminal *gem*‐dimethyl groups, leading to the corresponding chiral *β*‐methyl primary alcohols in good optical purity (51%–79% *ee*). The hydroxylation products can be further derivatized to chiral diols and lactones. Substitution of the highly conserved active site residue F169 to leucine increased the activity toward short and medium‐chain esters up to two‐fold. While the wildtype enzyme displayed very low conversion of long‐chain substrates, activity toward *n*‐dodecyl acetate could be increased 11‐fold by reducing the size of the tryptophan residue 60 situated in the putative substrate tunnel. Substitution of the peripheral I238 with valine increased activity regardless of the chain length of the substrate. Our results provide insight into the strong substrate dependency of the reported mutations and lay the groundwork for the establishment of a whole‐cell process for the regio‐ and stereoselective hydroxylation of linear and branched esters, leading to valuable bifunctionalized products. The deeper understanding gained from mutating key residues and the substrate acceptance of AlkB will guide future protein engineering campaigns.

## 1 INTRODUCTION

**FIGURE 1:**
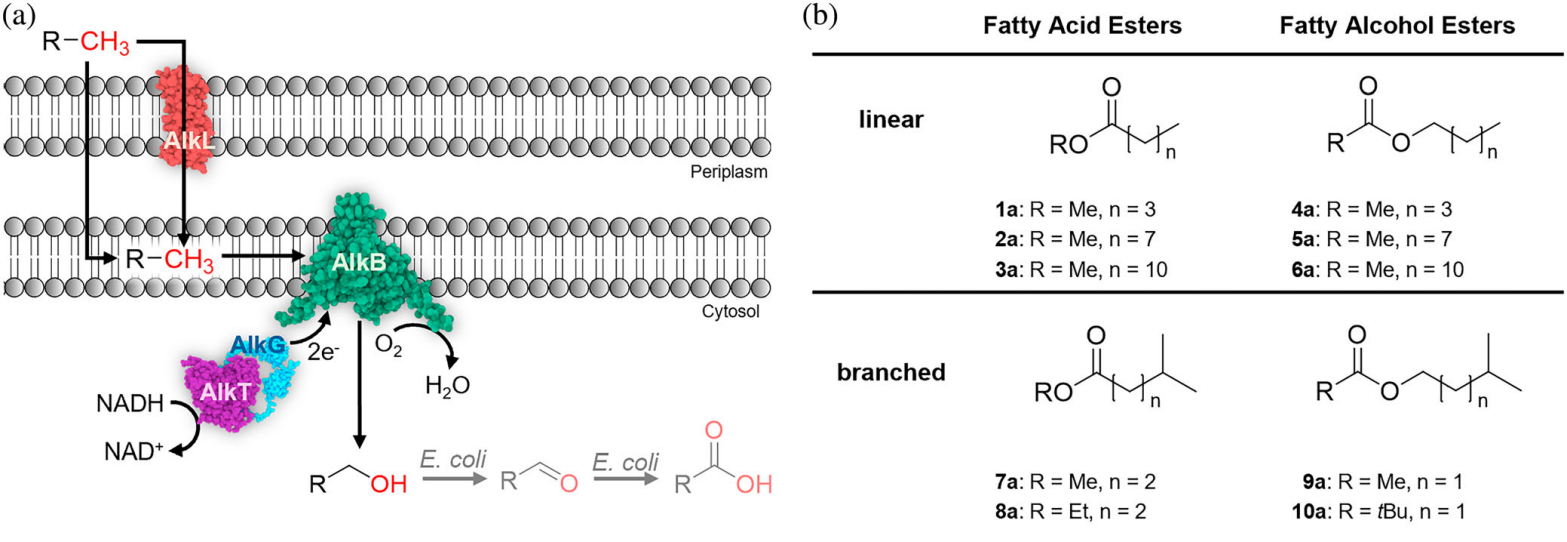
(a) Terminal hydroxylation of aliphatic compounds by alkane monooxygenases AlkB and potential oxidation of the resulting alcohol to the aldehyde and carboxylic acid by the host background. (b) Linear and branched esters investigated in this study.

The selective oxyfunctionalization of inherently inert C(sp^3^)–H bonds into more reactive C–O bonds is a highly important reaction in organic chemistry. It enables the synthesis of value‐added compounds such as alcohols, epoxides, aldehydes, ketones, and carboxylic acids (Goldman & Goldberg, 2004; Münch et al., 2021; Siedlecka, 2023; White, 2012; Wu et al., 2023), which can also be further functionalized to more complex molecules (Behmagham et al., 2024; Jelen & Tavčar, 2025; Ji et al., 2025; Leech & Lam, 2020; Moon et al., 2023). Challenges associated with the direct oxygenation of aliphatic C–H bonds include regio‐, chemo‐, and stereoselectivity, product yields, and sustainability (Goldman & Goldberg, 2004; Münch et al., 2021; Siedlecka, 2023; White, 2012; Wu et al., 2023). Nature has evolved numerous enzymes, typically relying on metal cofactors, that are capable of selectively oxyfunctionalizing C(sp^3^)–H bonds of various substrates (Austin & Groves, 2011; Dong et al., 2018; Münch et al., 2021; Wu et al., 2023). Many heme‐dependent cytochrome P450s (CYP450) target subterminal ω‐1 and ω‐2 positions, with only a few being specific for the terminal position (Ebrecht et al., 2025; Fiorentini et al., 2018; Funhoff et al., 2006; Hammerer et al., 2018; Honda Malca et al., 2012; Meinhold et al., 2006; Schultes et al., 2024). In contrast, the diiron non‐heme alkane monooxygenase AlkB exhibits exceptional regioselectivity for the terminal hydroxylation of linear aliphatic compounds. AlkB and its electron transfer accessory proteins were first isolated from the soil bacterium *Pseudomonas putida* GPo1 (PpAlkB; recently renamed to *Ectopseudomonas oleovorans*, formerly known as *Pseudomonas oleovorans*) (McKenna & Coon, 1970; Peterson et al., 1966; Peterson & Coon, 1968). Since then, various homologs from other soil and marine bacteria have been identified (Nie et al., 2011; Ratajczak et al., 1998; Smits et al., 2002; Van Beilen et al., 2002, 2004; Williams et al., 2021; Williams et al., 2022; Williams & Austin, 2022). AlkB is part of the *alk*‐operon, which enables bacteria to use *n*‐alkanes as their sole energy and carbon source. The operon comprises genes encoding the rubredoxin AlkG, which transfers electrons to AlkB and the reductase AlkT, which reduces rubredoxin AlkG (Figure 1a). In *Pseudomonas putida* GPo1, the *alk*‐operon, located on the OCT plasmid, also encodes the rubredoxin AlkF, thought to be a duplication of the inactive N‐terminal region of AlkG, whose function remains unknown (Kok et al., 1989). Additionally, the operon encodes for the alcohol dehydrogenase AlkJ, the aldehyde dehydrogenase AlkH, a fatty acid‐CoA ligase AlkK, and an outer membrane transporter AlkL (Owen et al., 1984; van Beilen et al., 2001; van Beilen, Wubbolts, & Witholt, 1994). In addition to linear medium‐chain hydrocarbons (C_6_ to C_12_), AlkB also acts on branched, cyclic, and aromatic compounds. Terminal olefin groups are typically converted to epoxides (May & Abbott, 1973; May et al., 1977; May & Katopodis, 1986; van Beilen, Kingma, & Witholt, 1994). The well‐studied PpAlkB has been used to hydroxylate esters of fatty acids (FAcE) and fatty alcohols (FAlE) for the production of polyester precursors (Julsing et al., 2012; Schrewe et al., 2011; Schrewe et al., 2014; van Nuland et al., 2016; van Nuland, de Vogel, Eggink, & Weusthuis, 2017; van Nuland, de Vogel, Scott, et al., 2017). Terminal oxyfunctionalization in conjunction with amination yields precursors for polyamides (Herzog et al., 2020; Ladkau et al., 2016; Schrewe et al., 2013). The α,ω‐oxyfunctionalized products also find applications in the pharmaceutical (Rivière et al., 2020), cosmetic (Kosmadaki & Katsambas, 2017), and agricultural industry (Wang et al., 2007).

The challenges associated with the kinetic characterization of membrane‐bound enzymes and the elucidation of their structures have hampered efforts to engineer them. In the case of AlkB, only recently the first structures of two homologs from *Fontimonas thermophila* (FtAlkB) were determined by cryo‐electron microscopy (cryo‐EM) (Chai et al., 2023; Guo et al., 2023). However, the absence of high‐throughput screening (HTS) assays for unnatural substrates has limited its engineering to random mutagenesis and selection based on the utilization of natural *n*‐alkane substrates (Koch et al., 2009; van Beilen et al., 2005).

In a recent study, we described the chemoselectivity of PpAlkB in the conversion of isoprenyl acetate, a precursor of the biobased monomer tulipalin A. While heme‐dependent fungal unspecific peroxygenases oxidized the *exo*‐methylene group to the epoxide, PpAlkB selectively catalyzed the allylic oxidation (Nigl et al., 2025). The fact that AlkB accepted this branched substrate prompted us to further investigate the acceptance of substrates with a larger, prochiral *gem*‐dimethyl function. AlkB is known to hydroxylate (ω‐1)‐methyl‐alkanes with a strong preference for the linear terminus (van Beilen, Kingma, & Witholt, 1994). We hypothesized that the hydroxylation activity could be directed toward the prochiral methyl groups of the alkyl chain by protecting the linear end with a small ester group. This would allow us to investigate, for the first time, the stereoselectivity of AlkB toward prochiral *gem*‐dimethyl groups and unlock access to chiral hydroxy acids, diols, and lactones. These chiral compounds present interesting building blocks not only for polymer production (Engel et al., 2019; Gubbels et al., 2018; Schaffer & Haas, 2014; Spanjers et al., 2016), but also for pharmaceuticals (Suzuki et al., 2004; Takahashi et al., 2012).

In this work, we aimed to shed light on the substrate scope, activity, and stereoselectivity of a homolog from *Marinobacter* sp. (M_AlkB). Therefore, we investigated the effect of selected point mutations in the active site, substrate tunnel, and membrane‐cytosol interface on the reactivity of M_AlkB toward a set of linear and branched esters of fatty acids and alcohols varying in the length of the alkyl chain (Figure 1b).

## 2 RESULTS AND DISCUSSION

### 2.1 Characterization of the alkane monooxygenase M_AlkB from *Marinobacter* sp.

Petroleum‐contaminated areas show an enrichment of *alkB* genes and proved to be a vast source of *alkB* sequences (Head et al., 2006; Vomberg & Klinner, 2000; Yang et al., 2015). Bacteria of the genus *Marinobacter* are widely found in diverse marine and saline environments (Cheng et al., 2025). They are also frequently enriched and isolated from hydrocarbon‐contaminated sites (Kostka et al., 2011; Li et al., 2026; Ruiz et al., 2016). These bacteria are known for their metabolic versatility and the ability to grow on alkanes, with most strains showing a preference for medium‐chain *n*‐alkanes ranging from C_6_ to C_8_ (Gauthier et al., 1992; Gunasekera et al., 2022; Yang et al., 2015). Here, we characterize an AlkB from *Marinobacter* sp., derived from a metagenome sample (Tully et al., 2018), and was shown to have 1.6 and 2.3‐fold higher hydroxylation activity compared to the well‐described PpAlkB toward *n*‐octane and isoprenyl acetate, respectively (Nigl et al., 2025). M_AlkB shares 78% sequence similarity with PpAlkB (Eggink et al., 1987; McKenna & Coon, 1970; Owen et al., 1984) and 66% and 51% similarity with the AlkBs from *Fontimonas thermophila* FtAlkB (PDB 8SBB, Guo et al., 2023) and FtAlkBG (PDB 8F6T, Chai et al., 2023), respectively, whose structures have been recently solved by cryo‐EM (Figure S2). *M_alkB* was expressed in *Escherichia coli* BL21 (DE3) under the control of the natural alkane‐inducible *P*_*alkB*_ promoter, originating from *P. putida* GPo1 OCT plasmid, encoded on the pCom10 plasmid (Smits et al., 2001) together with the redox partners *alkFGT* and the transporter *alkL* from *P. putida* GPo1 (Figure S7). An initial substrate screening showed that M_AlkB converted esters of different chain lengths (**1a**–**6a**) to their respective hydroxy products (**1b**–**6b**; Figure S11). Although the initial screening was performed over 24 h, product formation reached saturation within 4 h. Therefore, whole‐cell activity measurements were performed using a 4 h reaction time. The mass balances of the biotransformations were not fully closed which could be attributed to substrate or product volatility, hydrolysis, or losses during extraction steps. In our reaction system, loss of substrate or product due to their high volatility was difficult to prevent. However, this technical challenge could be addressed in future studies by using a biphasic system or a substrate reservoir, as previously reported (Hoschek et al., 2019; Schrewe et al., 2014; van Nuland, de Vogel, Eggink, & Weusthuis, 2017). We observed that the fatty acid methyl esters (FAcME) **1a**–**3a** were partially hydrolyzed to the respective acids **1**–**3** (Figure S11A–C), which prevented efficient quantification of the enzymatic activity for **1a** and **3a**. FAcME hydrolysis was previously reported and attributed to endogenous esterases from *E. coli* (Julsing et al., 2012; Kadisch et al., 2017; Schrewe et al., 2013; van Nuland et al., 2016). In whole‐cell reactions with the acetate esters **4a**–**6a**, less hydrolysis was observed (Figure S11D, E). The conversion by the wildtype (WT) PpAlkB and M_AlkB of the C_12_ chain esters **3a** and **6a** was very low. This follows the previously reported substrate preference for PpAlkB (Schrewe et al., 2011; van Beilen, Kingma, & Witholt, 1994; van Nuland et al., 2016). The substrate chain length reported here accepted by M_AlkB falls within the range of medium‐chain monooxygenases, consistent with the reported activity toward *n*‐octane and isoprenyl acetate (Nigl et al., 2025).

**FIGURE 2:**
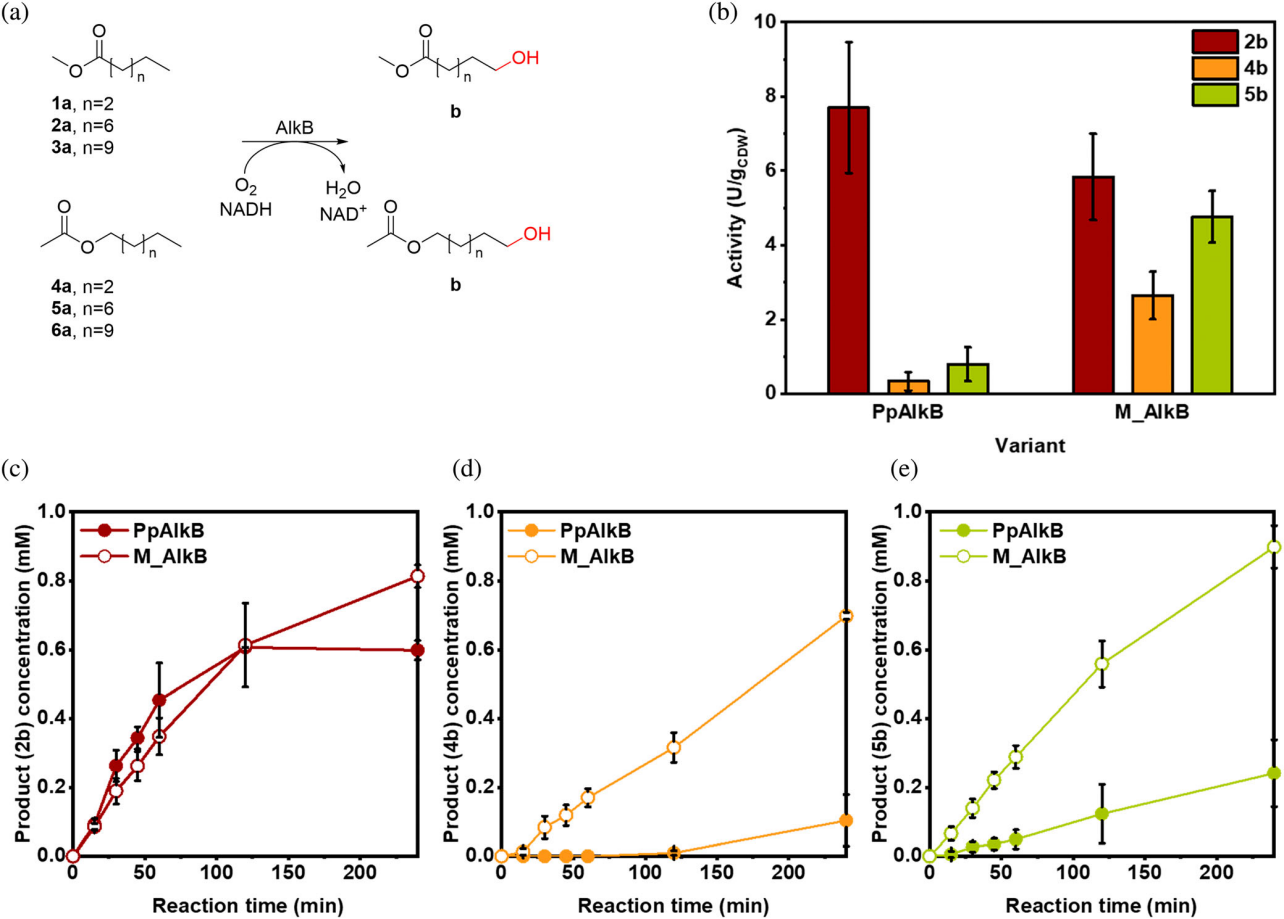
Biotransformation of linear FAcME and FAlAc by PpAlkB and M_AlkB. (a) Tested substrates and expected products; only residual (<2%) conversion of substrates **1a**, **3a**, and **6a** was observed. (b) Activity of PpAlkB and M_AlkB toward FAcME **2a** and FAlAc **4a** and **5a**. Time‐course of product formation for (c) **2b**, (d) **4b**, and (e) **5b** by PpAlkB (filled circles) and M_AlkB (empty circles). The reactions (300 μL) were performed with a cell density of 1 g_CDW_/L using 2 mM substrate (80 mM stock prepared in DMSO) at 25°C, shaking at 180 rpm for 4 h (mean ± SD; *n* = 3).

We determined the whole‐cell activity for the nonanoic acid methyl ester **2a** (van Nuland et al., 2016) and three fatty alcohol acetates (FAlAc) with varying chain lengths (**4a**, **5a**, and **6a**). M_AlkB showed comparable activity to PpAlkB toward **2a** (5.8 U/g_CDW_), whereas the activity toward medium‐chain acetates was up to 8‐fold higher, reaching 2.7 U/g_CDW_ and 4.8 U/g_CDW_ for **4a** and **5a**, respectively (Figure 2b–e). For **6a**, we observed residual conversion toward **6b** and overoxidized product **6d** (<3%) with both enzymes (Figure S11F). Interestingly, when comparing the whole‐cell oxygenation rates of methyl nonanoate **2a** with nonyl acetate **5a** by PpAlkB, the conversion of **2a** was significantly faster than that of **5a** (Figure 2C, E). In reactions with M_AlkB such a notable difference was not observed. Even though **2a** and **5a** have the same alkyl chain length and are sterically very similar, their hydrophobicity differs (Table S6). The logP values of **2a** and **5a** are 4.32 and 3.95, respectively. We assumed that hydrophobicity and partition coefficient of the substrates could influence the whole‐cell hydroxylation rates, especially when considering that the substrate is expected to enter the substrate tunnel leading to the enzyme active site via the lipid bilayer (Figure 1a) (Mikulska‐Ruminska et al., 2026). Amino acids forming the tunnel in M_AlkB and PpAlkB are almost identical; however, the enzymes' tunnels differ in the second‐shell residues that neighbor the tunnel‐forming residues (Figure S3C, D). This makes it very difficult to rationalize the difference in the acceptance of methyl alcohol esters and acetate esters by both membrane monooxygenases.

### 2.2 Enzyme engineering for increased activity toward linear esters

**FIGURE 3:**
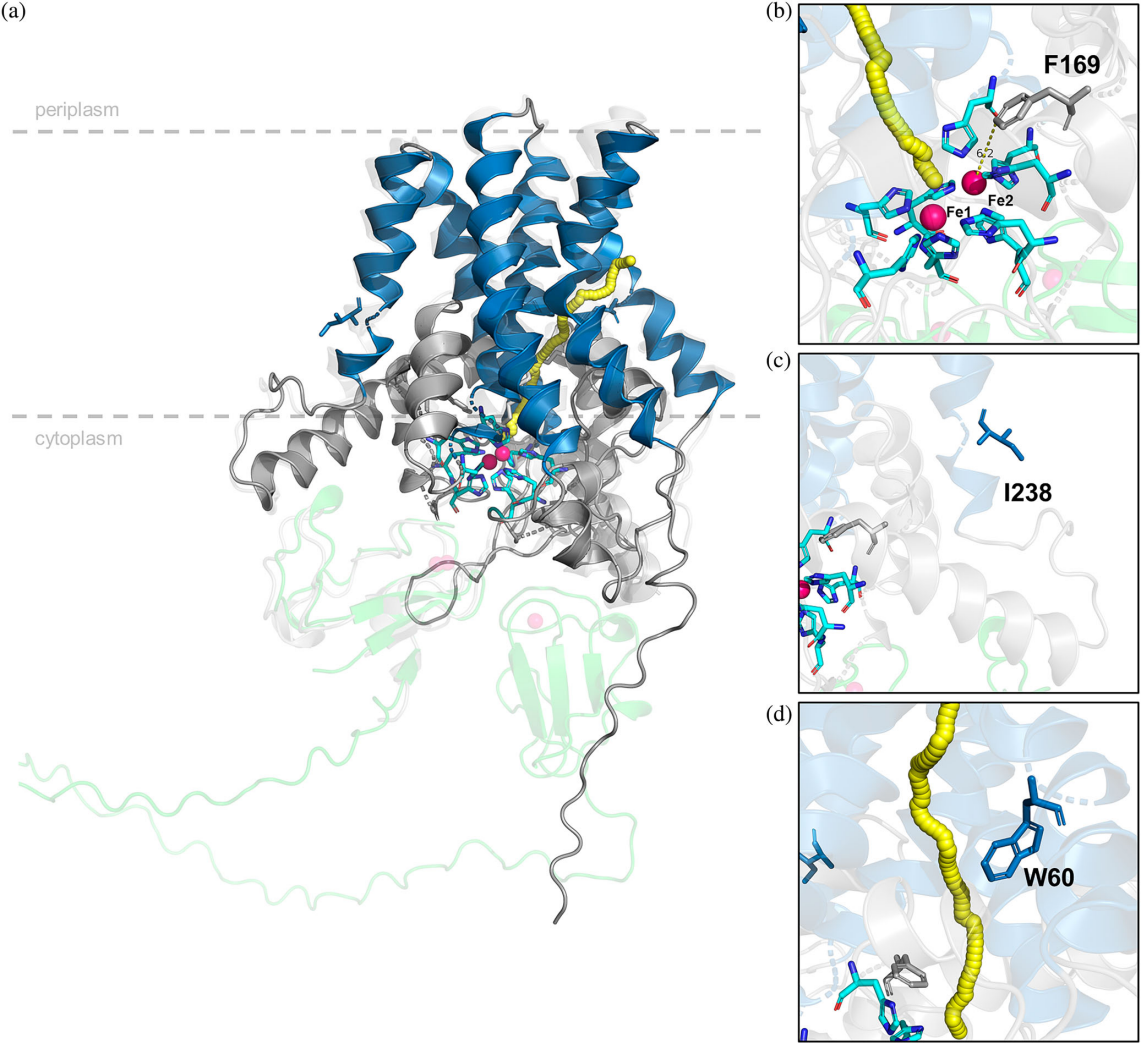
(a) AlphaFold 3 predicted structure of M_AlkB (dark gray) with highlighted transmembrane domains (dark blue), active site histidine residues (cyano) coordinating two iron atoms (pink), and substrate tunnel (yellow). The interaction with PpAlkG (UniProt P00272, light green) was predicted by AlphaFold 3. The predicted structure of the protein complex was aligned with FtAlkBG (PDB 8f6t, light gray). Amino Acid (AA) residues targeted for site‐directed mutagenesis: (b) F169 close to the active site, (c) I238 in the transmembrane domain facing the lipid bilayer, and (d) W60 at the entrance of the predicted substrate tunnel.

The selection assay which couples AlkB‐catalyzed terminal hydroxylation to cell growth was developed to screen enzyme variants that convert various chain‐length alkanes (Koch et al., 2009; van Beilen et al., 2005). However, this growth‐based assay cannot be used to screen for the conversion of the esters **1a**–**6a**, as we observed that these esters can be hydrolyzed by the cells, yielding primary alcohols or fatty acids. The hydrolysis products can be fed into the β‐oxidation pathway directly, rendering AlkB‐catalyzed terminal hydroxylation unnecessary for their utilization as a carbon source. Due to the lack of a proper HTS method, we focused on rational design. We investigated the effects of selected point mutations in the active site, the substrate tunnel, and the periphery. Figure 3 highlights the residues targeted by mutagenesis in the 3D structure of M_AlkB generated by AlphaFold 3 (Abramson et al., 2024). The putative substrate tunnel was predicted using CAVER Web 2.0 (Figure 3a) (Marques et al., 2025). The modeled enzyme consists of six transmembrane helices arranged in a slightly cone‐like shape, opening toward the cytosol. Helices 2, 4, and 6 are close to the predicted substrate tunnel, similar to those described for FtAlkB (Guo et al., 2023) and FtAlkBG (Chai et al., 2023). The predicted active site cavity is formed between the transmembrane helices and resembles a typical AlkB active site with the nine distinct, highly conserved histidines coordinating the two iron atoms essential for catalytic activity (Chai et al., 2023; Guo et al., 2023; van Beilen et al., 2005). The distance between two iron atoms in the active site is 5.9 Å and is in accordance with distances previously observed in the cryo‐EM structures of two homologs (5.4 Å for FtAlkB and 6.1 Å for FtAlkBG) (Chai et al., 2023; Guo et al., 2023). Although the typical distance in diiron enzymes spans from 2.7 to 4.1 Å (Fox et al., 2004; Jasniewski & Que, 2018; Merkx et al., 2001), in AlkB alkane monooxygenases, the two iron atoms are unusually far apart (>5 Å). Also, no apparent bridging ligands such as carboxylate‐containing residues, glutamate or aspartate, were observed between the iron atoms (Reinhardt et al., 2025), in contrast to the bridging motifs typical for other diiron‐containing enzymes (Caldas Nogueira et al., 2021; Jasniewski & Que, 2018). The unusual architecture of the AlkB active site still raises questions regarding the reaction mechanism (Austin et al., 2008; Chai et al., 2023; Groves et al., 2023; Guo et al., 2023; Mikulska‐Ruminska et al., 2026; Reinhardt et al., 2025; Wang & Liu, 2024). For example, Groves et al. recently proposed a relay mechanism in which the two iron ions play distinct roles, taking the long distance between them into account (Figure S6) (Groves et al., 2023).

**FIGURE 4:**
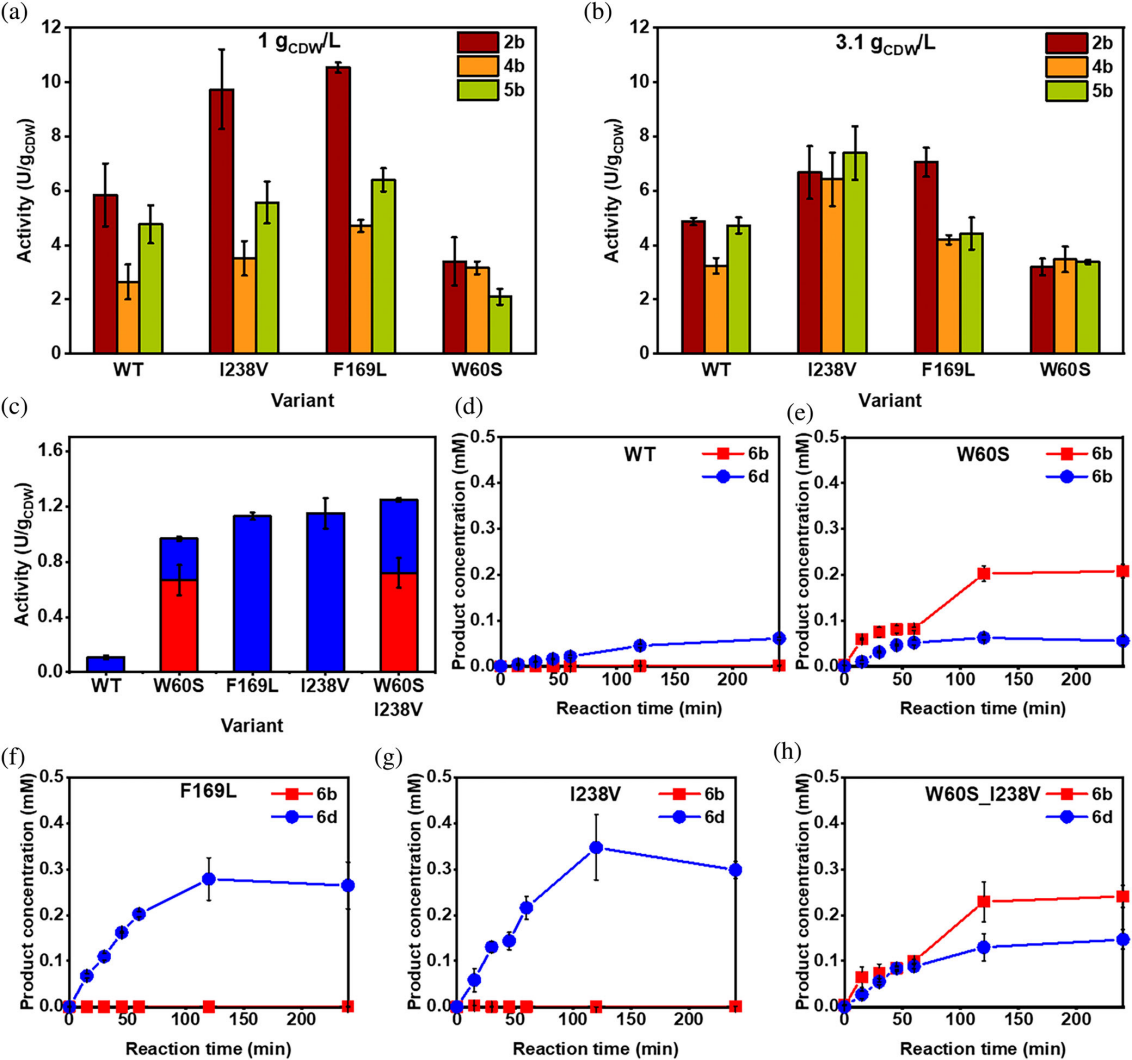
Hydroxylation activities of M_AlkB wildtype (WT) and variants using linear esters **2a**, **4a**, and **5a** at (a) 1 g_CDW_/L and (b) 3.1 g_CDW_/L. (c) Activities and (d)–(h) time‐course of M_AlkB wildtype (WT), W60S, F169L, I238V and W60S_I238V with **6a** as substrate at 3.1 g_CDW_/L. The reactions (300 μL) were performed using 2 mM substrate (80 mM stock prepared in DMSO), at 25°C, shaking 180 rpm for 4 h (mean ± SD; *n* = 3).

To improve the enzyme's activity, several point mutations were tested (Figure 3b–d). Residue I238 is located close to the periplasm in the fifth predicted transmembrane helix, distant from the active site (Figure 3c). The analog substitution of I233 to valine in PpAlkB was identified in a random mutagenesis‐based directed evolution as part of a triple mutant, which showed improved activity toward butane and pentane (Koch et al., 2009). M_AlkB I238V showed higher activity toward *n‐*octane and isoprenyl acetate (Nigl et al., 2025). As shown in Figure 4a, at a cell density of 1 g_CDW_/L, the mutation I238V increased the hydroxylation activity toward **2a**, **4a**, and **5a**, resulting in 9.7, 3.5, and 5.6 U/g_CDW_, respectively. Increasing the cell density to 3.1 g_CDW_/L still led to higher activity compared to the wildtype enzyme, reaching 6.7, 6.4, and 7.4 U/g_CDW_ for **2a**, **4a**, and **5a**, respectively (Figure 4b). At higher cell density, product formation rate for acetates increased up to 2‐fold, while for methyl ester **2a**, the improvement was only 1.37‐fold. Overall, this M_AlkB variant led to the highest product yields obtained after 2 h, resulting in 0.92 mM **2b** (Table S18). These results indicate that the I238V mutation indeed increases activity of AlkB toward linear esters in the whole‐cell biocatalysts, independent of cell density and substrate chain length, which confirms results from previous engineering studies (Koch et al., 2009; Nigl et al., 2025).

The residue F169 is situated in the active site, being 6.2 Å distant from the second Fe atom (Fe2 in Figure 3b). Its substitution to leucine was recently reported to improve the activity toward isoprenyl acetate (Nigl et al., 2025). This residue is highly conserved among homologs (99% F, Table S1). It is, therefore, interesting that a change to leucine led to improved activity for **2a**, with up to a 1.8‐fold increase reaching 10.5 U/g_CDW_. In contrast to I238V, the activity of F169L strongly depended on cell density, with a pronounced increase in activity at lower cell densities (1 g_CDW_/L). The dependence of enzyme activity on cell density could arise from transport limitations, substrate availability, or metabolic background. We expect that these are comparable between WT and the tested mutants. However, oxygen availability at different cell densities might affect the reaction rates of WT and selected mutants. For whole cell biocatalytic oxygenation performed in *E. coli* at higher cell density (>2 g_CDW_/L), it is known that oxygen mass transfer can be a limiting factor (Baldwin & Woodley, 2006). As reported biotransformations herein were performed using a cell density of either 1 or 3.1 g_CDW_/L, we assume that the cells face different oxygen availability, which can, in turn, affect the enzymatic activity.

In other AlkB homologs, the substitution of the tryptophan located at the entrance of the substrate tunnel (Figure 3d) in the second predicted transmembrane helix (W55 in PpAlkB, W62 in FtAlkB, W60 in M_AlkB) to a smaller amino acid, such as serine or valine, shifted the substrate acceptance of the monooxygenases toward longer substrates (> C_12_) (Guo et al., 2023; van Beilen et al., 2005). The position of this tryptophan in the predicted tunnel leading from the membrane as a possible substrate reservoir to the active site suggests that this residue might act as a gate‐keeper for long‐chain substrates (Guo et al., 2023; van Beilen et al., 2005). For the medium‐chain esters **2a**, **4a**, and **5a**, the analogous mutation W60S in *M_alkB* did not lead to improved activity, but rather to a decreased hydroxylation rate, more pronounced at a lower cell density. However, this residue was tested, knowing that the conversion of longer chain substrates might be enhanced. Although variants I238V and F169L showed only traces of **6b** (<0.002 mM), we observed up to a 10‐fold increase in the product formation rate of **6d** (1.15 and 1.13 U/g_CDW_, respectively) compared to WT (0.11 U/g_CDW_) (Figure 4c). M_AlkB W60S produced a mixture of **6b** and **6d**, reaching an overall activity of 0.97 U/g_CDW_ after 2 h at 3.1 g_CDW_/L (Figure 4e, Table S18). In further testing, the M_AlkB double variant W60S_I238V, the initial activity of 1.25 U/g_CDW_ yielded 0.23 mM **6b** and 0.13 **6d** after 2 h using 3.1 g_CDW_/L cell density (Figure 4h, Table S18).

Our results highlight that the knowledge gained by rational engineering can be transferred among AlkB homologs and that the effect on the activity toward alkanes (Guo et al., 2023; Koch et al., 2009; Nigl et al., 2025; van Beilen et al., 2005) can also influence the activity toward esters. For instance, the mutation I238V in *M_alkB* seems to improve the catalytic efficiency of the whole‐cell biocatalyst independent of the substrate. The molecular effect of the peripheral substitution I238V remains to be clarified, being 22.4 Å from Fe1 and 25.1 Å from Fe2 of the active site. Nevertheless, I238 is only 8.2 Å distant from a flexible loop closely situated to R215 and E232, which potentially play a role in the binding of AlkG (Groves et al., 2023; Mikulska‐Ruminska et al., 2026). The limited acceptance of the long‐chain substrate **6a** could be enhanced via the single mutations F169L and I238V, which mainly yielded the overoxidized product **6d**. The variant W60S and the double mutation W60S_I238V, however, showed increased formation of the hydroxylated product **6b** with the initial overall activity of 0.97 U/g_CDW_ and 1.25 U/g_CDW_, respectively. The increased hydroxylation rate for W60S variants indicates improved substrate access through the tunnel, further underlining the potential function of W60 as a gatekeeper in the substrate tunnel (Guo et al., 2023; van Beilen et al., 2005). Overall, our rate improvement of the whole‐cell biocatalysts by enzyme engineering resulted in up to 11‐fold improvements and is similar to fold‐changes obtained previously, reporting 2.6‐fold improved butane conversion (Koch et al., 2009) and 6‐fold improved isoprenyl acetate conversion (Nigl et al., 2025). These results demonstrate that the protein engineering of M_AlkB increased the conversion of linear C_5_–C_12_ esters, representing a new starting point to produce ω‐hydroxy acetates, diols and hydroxy acids.

### 2.3 Activity and stereoselectivity of M_AlkB WT and mutants toward branched esters

**FIGURE 5:**
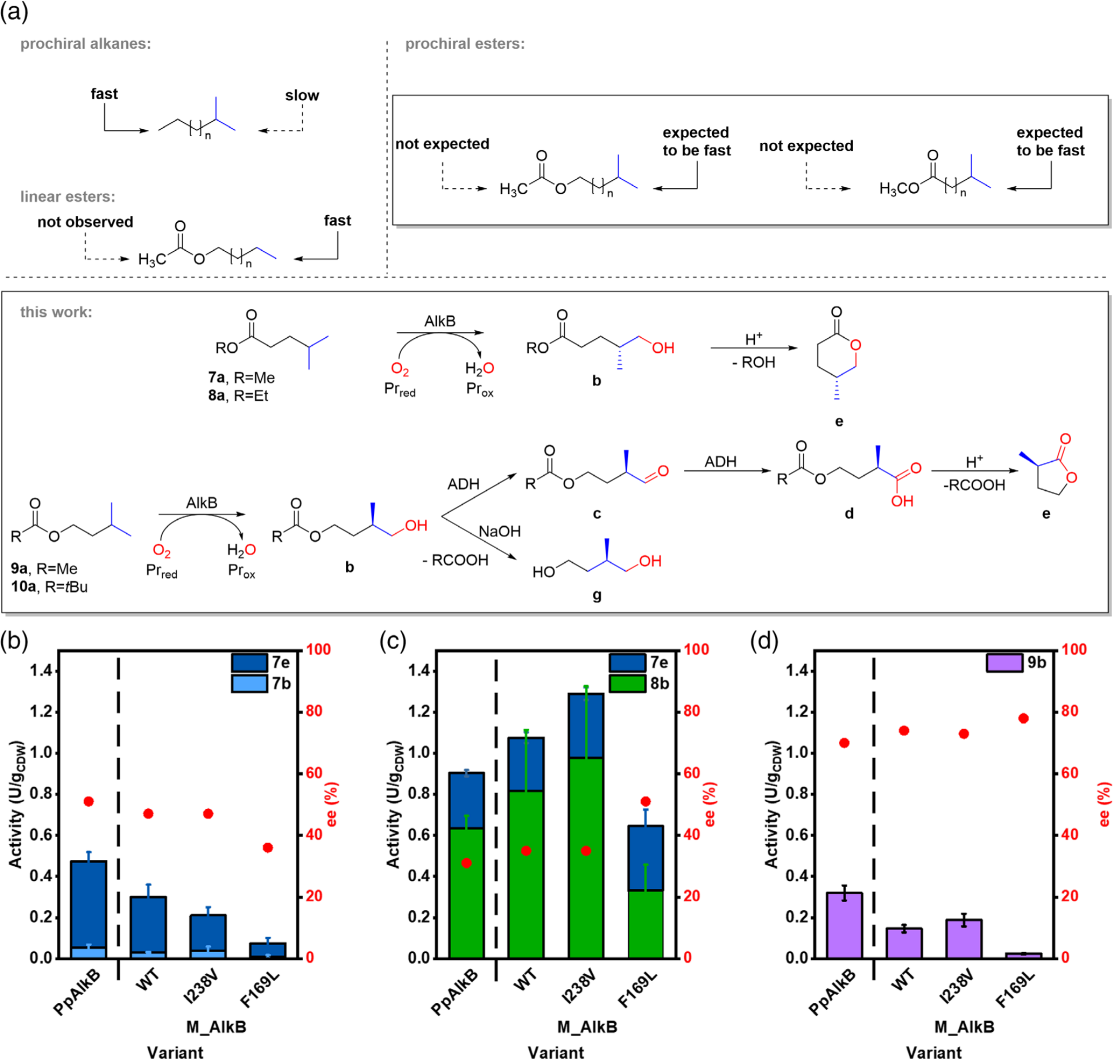
(a) Rationale behind the route toward optically pure hydroxy acids, diols, and lactones via protection of the linear terminus with an ester group leading to stereoselective terminal hydroxylation of the prochiral *gem*‐dimethyl group by AlkB. Hydroxylation activity and stereoselectivity of PpAlkB and M_AlkB variants performed at 3.1 g_CDW_/L toward the branched esters (b) **7a**, (c) **8a**, and (d) **9a**. The reactions (1 mL) were performed with a cell density of 3.1 g_CDW_/L using 2 mM substrate (80 mM stock prepared in DMSO or EtOH), at 25°C, shaking 180 rpm, for 24 h and data are represented as arithmetic mean ± SD (*n* = 3).

AlkB monooxygenase has been previously reported to catalyze the allylic oxidation of sterically hindered substrates (Table S19), such as isobutene (Engel et al., 2015) and isoprenyl acetate (Nigl et al., 2025). We hypothesized that structurally similar substrates with a terminal *gem‐*dimethyl group should also be converted, even though the steric hindrance is higher than that of the *exo*‐olefin group. Due to the prochiral nature of the *gem‐*dimethyl group, hydroxylation of one of the methyl groups would give rise to a chiral primary alcohol. May et al. reported the formation of enantiomerically enriched (*R*)‐epoxides from 1‐alkenes by PpAlkB (May & Abbott, 1972, 1973). In the conversion of terminally branched dimethyl alkanes, such as 2‐methyl octane (**11a**), PpAlkB strongly preferred hydroxylation of the linear terminus (van Beilen, Kingma, & Witholt, 1994). Indeed, the selectivity of M_AlkB for the hydroxylation of the linear‐ over the branched end was 36‐fold, leading to **11b** with only traces of chiral **11c** observed (Figure S14, Table S17). van Nuland et al. showed that PpAlkB does not act on terminal methyl ester or acetyl ester groups (van Nuland et al., 2016; van Nuland, de Vogel, Scott, et al., 2017). We hypothesized that in an ester composed of a germinal dimethyl end and a methyl ester or acetyl ester end, the activity of AlkB would be directed to the prochiral group, albeit with lower activity. This would allow us to determine the capacity of AlkB to discriminate between the two methyl groups (Figure 5a). To confirm our hypothesis, we investigated the hydroxylation of several prochiral esters **7a** to **10a**, varying in their chain length and the protective ester group (Figure 5a). The terminal hydroxylation of the branched FAcME **7a** would result in the chiral hydroxy fatty acid ester **7b**, which can easily be converted to the corresponding lactone **7e**. Similarly, terminal hydroxylation of the ethyl ester **8a** would result in the chiral ω‐hydroxy fatty acid ester **8b**, which can be lactonized to **7e** (Figure 5a). On the other hand, terminal hydroxylation of the FAlE **9a** and **10a** would form the corresponding monoester of 2‐methyl‐1,4‐butanediol (**9b**) (Figure 5a). We were pleased to see the formation of the corresponding hydroxy products **7b** and **9b** starting from prochiral **7a** and **9a**, respectively (Figures S15 and S16). The formation of **7b** was further confirmed by the presence of the corresponding lactone **7e**. The hydroxylation of **9a** to **9b** was confirmed by comparison with an authentic standard produced by the esterification of the diol **9g** using a *Pseudomonas cepacia* lipase. Initial testing in 1.5 mL vials at a cell density of 1 g_CDW_/L led to very low conversions of the sterically demanding substrates. Henceforth, the biotransformations were conducted at 3.1 g_CDW_/L in sealed glass vials of 20 mL, leading to increased product formation. To facilitate dissolution of the hydrophobic substrates, stocks were prepared in water‐miscible cosolvents, ethanol or DMSO (both 2.5% (v/v)). Interestingly, in biotransformations with DMSO as a cosolvent, we observed further oxidation of the alcohol **9b** to the corresponding aldehyde **9c** and carboxylic acid **9d**, which was further lactonized to **9e** (74% *ee*) under acidic conditions (Figures S16B and S18C, D). The overoxidation is probably caused by alcohol dehydrogenases present in *E. coli* (van Nuland, de Vogel, Eggink, & Weusthuis, 2017). On the contrary, no overoxidation was observed when ethanol was used as a cosolvent.

Under optimized reaction conditions, M_AlkB WT showed an activity of 0.27 U/g_CDW_ and 0.15 U/g_CDW_ for the hydroxylation of **7a** and **9a**, which is 1.6‐fold and 2.1‐fold lower compared to PpAlkB (0.42 and 0.32 U/g_CDW_, respectively). Interestingly, by extending the methyl to an ethyl moiety in **8a**, the activity was higher, reaching 1.08 U/g_CDW_ for M_AlkB and 0.90 U/g_CDW_ for PpAlkB. This could be attributed to the higher logP of **8a** (2.44), compared to **7a** (2.08). Even though an even longer alkyl chain of the alcohol moiety of the ester of branched carboxylic acids might improve activity (van Nuland et al., 2016), a longer group than ethyl might be itself hydroxylated by the enzyme, leading to product mixtures. Therefore, we considered further increasing the size of the ester moiety as a strategy to increase the activity and selectivity. However, the ester **10a** containing a *tert*‐butyl group was barely converted (<2%) (Figure S19), indicating that M_AlkB is rather limited in the conversion of bulky substrates.

Further more, we investigated the influence on the activity and selectivity of several M_AlkB mutants, which increased the enzymatic activity with linear esters (Figure 4). The M_AlkB variant I238V was slightly less active toward **7a**, reaching 0.17 U/g_CDW_, and was more active toward **8a**, reaching 1.29 U/g_CDW_. For **9a**, it displayed comparable activity with a WT of 0.19 U/g_CDW_. Interestingly, the variant M_AlkB F169L, which showed higher activity with the sterically demanding isoprenyl acetate (Nigl et al., 2025) and linear esters, showed only 0.06 U/g_CDW_ for **7a**, while it reached 0.64 U/g_CDW_ for **8a**, still less than the wildtype enzyme. While the phenylalanine to leucine substitution changes size and hydrophobicity, this observed effect on the activity on the prochiral substrates is difficult to rationalize. Overall, none of the characterized variants from this study increased the activity toward esters having a *gem*‐dimethyl group. The activity of M_AlkB and its variants was about 20‐fold lower toward the sterically hindered esters compared to the conversion of linear esters of the same length, with activities ranging from 0.02 to 1.29 U/g_CDW_. Although activities for all tested AlkB variants were low, using PpAlkB, we produced 0.54 mM of lactone **7e** (starting from 2 mM **7a**) after 24 h (Table 1). The generally lower level of activities toward the branched substrates indicates a specific limiting factor which is not alleviated by the mutagenesis.

**TABLE 1:** Summary of screening of AlkB WT and variants with the prochiral ester **7a** and **8a** at 3.1 g_CDW_/L (mean ± SD; *n* = 3).

Substrate	Enzyme	Variant	Activity (U/g_CDW_)	Activity^a^ (U/g_CDW_)	7e (mM)^b^	7e *ee* (%)
**7a**	PpAlkB	WT	0.05 ± 0.01^c^	0.42 ± 0.05	0.54 ± 0.07	51 (*R*)
	M_AlkB	WT	0.031 ± 0.001^c^	0.27 ± 0.06	0.37 ± 0.04	47 (*R*)
		I238V	0.04 ± 0.02^c^	0.17 ± 0.04	0.18 ± 0.03	47 (*R*)
		F169L	0.010 ± 0.007^c^	0.06 ± 0.03	0.04 ± 0.001	36 (*R*)
**8a**	PpAlkB	WT	0.63 ± 0.06^d^	0.27 ± 0.02	0.28 ± 0.05	31 (*R*)
	M_AlkB	WT	0.82 ± 0.30^d^	0.26 ± 0.03	0.31 ± 0.03	35 (*R*)
		I238V	0.98 ± 0.35^d^	0.31 ± 0.03	0.27 ± 0.01	35 (*R*)
		F169L	0.33 ± 0.12^d^	0.31 ± 0.08	0.25 ± 0.03	51 (*R*)

^a^ Product formation rate of **7e**.

^b^ Obtained after 24 h.

^c^ Product formation rate of **7b**.

^d^ Product formation rate of **8b**.

PpAlkB and M_AlkB displayed similar % *ee* (51 and 47, respectively) with both preferentially forming (*R*)‐**7e** lactone (Figure S17 and Table 1). The enantiomeric excess of the lactone **7e** formed by the M_AlkB variants I238V and F169L was comparable with 47% and 36% *ee*, respectively. We were wondering whether prolonging the methyl ester to an ethyl group affects the enantioselectivity. Reactions with **8a** still resulted in the preferred formation of the (*R*)‐enantiomer. However, compared to the methyl ester **7a**, the *ee* decreased to 35% for the WT, while for the F169L variant, the *ee* increased to 51% (Table 1). Higher stereoselectivity was observed for M_AlkB‐catalyzed hydroxylation of the branched FAlAc **9a**, yielding 79% *ee* of (*R*)**‐9b**. Base‐catalyzed hydrolysis of **9b** to **9g** slightly reduced the *ee* to 74%, which is similar to results obtained with PpAlkB (81% *ee* (*R*)‐**9b** and 70% *ee* (*R*)‐**9g**). All tested variants showed similar stereoselectivity in the hydroxylation of **9a** (73%–78% *ee*) compared to the WT. Tables 1, and 2 provide a detailed overview of the activity and stereoselectivity of PpAlkB and M_AlkB wildtype and variants with the branched esters **7a**, **8a**, and **9a**.

**TABLE 2:** Summary of screening of AlkB WT and variants with the prochiral ester **9a** at 3.1 g_CDW_/L (mean ± SD; *n* = 3).

Substrate	Enzyme	Variant	Activity (U/g_CDW_)	9b (mM)^a^	9b *ee* (%)	9 g *ee* (%)
**9a**	PpAlkB	WT	0.32 ± 0.04	0.59 ± 0.02	81 (*R*)	70 (*R*)
	M_AlkB	WT	0.15 ± 0.02	0.22 ± 0.02	79 (*R*)	74 (*R*)
		I238V	0.19 ± 0.03	0.34 ± 0.01	79 (*R*)	73 (*R*)
		F169L	0.02 ± 0.01	0.04 ± 0.01	67 (*R*)	78 (*R*)

^a^ Obtained after 24 h.

Stereoselectivity of PpAlkB was previously shown only for epoxidation of terminal alkenes with *pro*‐*R* stereoselectivity (May & Abbott, 1972, 1973), whereas its capacity to discriminate between different alkyl groups of branched hydrocarbons remained unknown. By using a protection strategy of acetate or ester group for the linear C‐terminus, we suppressed the competing hydroxylation of the linear terminus of the substrate and directed the hydroxylating activity toward the prochiral group. PpAlkB and M_AlkB are *pro*‐*R* selective in the hydroxylation of the *gem‐*dimethyl group, leading to the production of chiral lactone **7e** and chiral diol **9g**. The chiral synthon **7e** is used for the synthesis of active enantiomers of the pheromones of pine sawflies, important for pesticide control (Wang et al., 2007). Interestingly, a previously reported chemosynthetic synthesis of (*R*)‐**7e** was based on functionalizing common natural monoterpenoid L‐(−)‐menthol (Ishmuratov et al., 2004). Our results demonstrate a new biocatalytic route starting from cheap branched esters toward chiral diols and lactones that can be further derivatized into a vast number of chiral building blocks. The obtained results indicated that fatty acid alkyl esters showed better activity than the esters of medium‐chain length alcohols. This could be attributed to the difference in the steric hindrance of the methyl ester or acetate group or the difference in hydrophobicity between both. Recent studies proposed the reaction mechanism of alkane monooxygenases for terminal hydroxylation of linear alkanes (Chai et al., 2023; Guo et al., 2023). The molecular mechanism for the stereoselective hydroxylation of prochiral esters is still unknown. Our results indicate two possible binding modes, with *pro*‐*R* being preferential. Future efforts will be directed to the identification of amino acid residues directing activity and selectivity toward these substrates and further improving the activity of the monooxygenase by enzyme engineering.

## 3 CONCLUSION

Alkane monooxygenases show unique selectivity in the terminal hydroxylation of a wide range of substrates. The lack of accurate structural models has, so far, hampered the engineering of these interesting biocatalysts. Herein, we demonstrated the rational engineering of M_AlkB, isolated from a metagenomic dataset, to improve enzymatic activity toward the synthesis of valuable hydroxylated aliphatic esters. With these results, we gained insight into the substrate preference and stereoselectivity of M_AlkB, an enzyme that appears to be particularly interesting for the conversion of short to medium‐chain esters. Using a protection strategy to direct hydroxylation of the branched C‐terminus enabled us, for the first time, to characterize the stereoselectivity of alkane monooxygenase toward prochiral terminal *gem*‐dimethyl group. This provided a new enzymatic reaction pathway for the synthesis of chiral lactones, hydroxy acids, and diols in good optical purity. We expect that the herein presented results on the substrate scope and the strong substrate dependency of the reported mutations of M_AlkB will guide further enzyme engineering studies of bacterial membrane‐bound alkane monooxygenases.

## 4 MATERIALS AND METHODS

### 4.1 Chemicals

If not stated otherwise, all commercially available chemicals and compounds used in this work were purchased either from Merck/Sigma‐Aldrich (Darmstadt, Germany), TCI chemicals (Tokyo, Japan), or Carl Roth (Karlsruhe, Germany), usually in the highest purity available. The standard methyl 9‐hydroxynonanoate **2b** was purchased from Ambeed (Arlington Heights, IL, USA). 5‐methyltetrahydropyran‐2‐one **7e** and (5*R*)‐tetrahydro‐5‐methyl‐2H‐pyran‐2‐one (*R*)‐**7e** were purchased from Angene Chemical (Nacharam, India), 2‐methylbutane‐1,4‐diol **9g** was purchased from Enamine Ltd. (Kyiv, Ukraine), and (2*R*)‐2‐methylbutane‐1,4‐diol (*R*)‐**9g** was purchased from ABCR (Karlsruhe, Germany).

### 4.2 Cloning of the *alk*‐operon

The plasmid for the expression of *M_alkB* from *Marinobacter* sp. (M_AlkB; NCBI protein: MAB50652.1) was previously prepared by cloning synthetic DNA fragments from IDT into the *pPpalkBFGTL* vector via Gibson Assembly, replacing the *alkB*, while leaving the electron transfer system *alkFGT* from *P. putida* GPo1 intact, as these redox partners proved to be well accepted by the homolog from *Marinobacter* sp. (Nigl et al., 2025). Although rubredoxin AlkG from *P. putida* GPo1 shares 52% sequence identity with rubredoxin originating from the same metagenomic sample (NCBI ID MAB50653.1) as the sequence of M_AlkB, these redox mediators share a highly conserved three‐dimensional structure (Figure S5). Single and combinatorial mutants of *M_alkB* were either available in‐house (Nigl et al., 2025) or generated by site‐directed mutagenesis (SDM) (Table S8). Successful mutagenesis was verified by Sanger sequencing (Microsynth, Vienna, Austria). Table S7 lists all the strains used in this study.

### 4.3 Heterologous protein production and whole‐cell biotransformations

*E. coli* BL21(DE3) cells were transformed with the p*M_alkB*(mutant)*FGTL* expression constructs based on the pCom10 backbone, where the expression of the *alkBFGTL* operon is under the control of the natural alkane‐inducible promoter *P*_*alkB*_ (Smits et al., 2001). All substrates were tested in whole‐cell biotransformations using *E. coli* BL21(DE3) expressing *M_alkB*(mutant)*FGTL*, similar to previous work (Nigl et al., 2025). Briefly, a single colony of freshly plated *E. coli* containing the respective construct was used to inoculate lysogeny broth (LB; 5 mL) and incubated overnight at 37°C with constant agitation (120 rpm). Adapted M9 minimal media (M9MM; 20 mL) (Nigl et al., 2025) was inoculated with the seed culture (200 μL) and incubated at 30°C for 18–20 h. For heterologous gene expression, M9MM (200 mL) was inoculated to an OD_600_ of 0.15 with the respective preculture, grown at 30°C until an OD_600_ of 0.4 to 0.5, and induced by adding 0.05% (v/v) dicyclopropyl ketone (DCPK). After 4 h of expression at 30°C, the cells were harvested (4400 × *g*, 4°C, 15 min) and resuspended in resting cell (RC) buffer (50 mM KPi, pH 7.4, 2 mM MgSO_4_, 1% glucose) to an OD_600_ of 3.2 or 10 (corresponding to 1 g_CDW_/L, and 3.1 g_CDW_/L, respectively). The reaction was initiated by adding 2 mM of substrate (from an 80 mM stock solution in EtOH or DMSO) to the RCs and performed at 25°C, 180 rpm, with a total volume of either 300 μL in 1.5 mL or 1 mL in 20 mL tightly sealed glass vials. The initial screening of substrates was performed for 24 h, while for the quantification, the reactions were performed for 4 and 24 h for linear and branched esters, respectively. To avoid losses of volatile compounds, for each sampling point, a separate reaction (300 μL or 1 mL) was prepared from a master mix. Reaction samples (250 μL) were taken and quenched by adding 2 M HCl (25 μL) and stored at −20°C until further analysis.

### 4.4 Gas chromatography (GC) analysis

To analyze biotransformation samples, the quenched samples (275 μL) were extracted with EtOAc (250 μL) containing 1 mM methyl benzoate as internal standard (ISTD) by vigorous shaking for 1 min. After phase separation by centrifugation (16,000 × *g*, 4°C, 5 min), the organic layer was dried over Na_2_SO_4_, and the extract (200 μL) was directly subjected to GC analysis. To obtain the lactone **9e**, the biotransformation sample (300 μL) was quenched with 2 M HCl (30 μL), centrifuged (16,000 × *g*, 4°C, 5 min), and the supernatant was incubated for 1 h at 80°C, 200 rpm in a thermoshaker. A total of 275 μL was then extracted with EtOAc (250 μL) containing ISTD as described. Chiral diols were analyzed by extracting non‐quenched biotransformation samples (500 μL) with MTBE (250 μL). After the phases were separated by centrifugation (16,000 × *g*, 4°C, 5 min), the organic phase was mixed with 10 M NaOH (2.5 μL), and the sample was incubated at 40°C and 900 rpm for 24 h in a thermoshaker. The sample was dried over Na_2_SO_4_ and directly analyzed by GC.

A GC coupled to a mass spectrometer (GC–MS) was used for qualitative analysis of the reactions and product identification. The measurements were performed on a Shimadzu GC–MS‐QP2010 SE (Shimadzu, Kyoto, Japan) equipped with a Zebron ZB‐5Plus GC column (30 m × 0.25 mm × 0.25 μm, Phenomenex (Torrance, CA, USA)), using the method presented in Table S9. For quantitative analysis, the extracted reaction samples were analyzed on a GC with a flame ionization detector (GC‐FID) from Shimadzu Nexis GC‐2030 equipped with a Zebron ZB‐5 capillary column (30 m × 0.25 mm × 0.25 μm) from Phenomenex (Torrance, CA, USA). Details on the GC‐FID methods used for achiral and chiral analysis are provided in Tables S10–S12. Due to the lack of authentic standards for **1b** and **3b**, their hydroxy products and overoxidized products were only tentatively identified by GC–MS. The concentrations of the analytes were calculated by external calibration curves generated by measuring samples with known concentrations of the pure compounds extracted from the RC buffer and treated equally to reaction samples. ISTD (1 mM methyl benzoate) was added during the extraction procedure to correct for variability in extraction, injection and instrument response. Enzymatic activities (U/g_CDW_) were obtained from biological triplicates and calculated as μmol of product(s) formed per min and g_CDW_. The data is represented as the arithmetic mean ± standard deviation (SD).

Chiral compounds were analyzed by GC‐FID (Shimadzu Nexis GC‐2030, Shimadzu, Kyoto, Japan) using either a Hydrodex *β*TBDAc (50 m × 0.25 mm × 0.25 μm, Macherey‐Nagel, GmbH & Co. KG, Düren, Germany) or a Hydrodex *β*TBDM (25 m × 0.25 mm × 0.25 μm, Macherey‐Nagel, GmbH & Co. KG, Düren, Germany) column. Tables S11 and S12 provide detailed descriptions of the methods used.

## AUTHOR CONTRIBUTIONS

**Jelena Spasic:** Conceptualization; investigation; writing – original draft; funding acquisition. **Andrea Nigl:** Conceptualization; investigation; writing – original draft. **Huijin Cheon:** Conceptualization; investigation; writing – original draft. **Christine L. Kaiserer:** Investigation; writing – review and editing. **Stela Galušić:** Investigation; writing – review and editing. **Elske van der Pol:** Investigation; writing – review and editing. **Lenny Malihan‐Yap:** Investigation; writing – review and editing. **Jin‐Byung Park:** Conceptualization; supervision; writing – review and editing; funding acquisition. **Robert Kourist:** Conceptualization; investigation; funding acquisition; supervision; resources; writing – original draft.

## CONFLICT OF INTEREST STATEMENT

The authors declare no conflicts of interest.

## Supporting information


**Data S1:** Supporting Information.

## Data Availability

The data that support the findings of this study are available from the corresponding author upon reasonable request.
